# Determinants of quality prostate cancer survivorship care across the primary and specialty care interface: Lessons from the Veterans Health Administration

**DOI:** 10.1002/cam4.2106

**Published:** 2019-04-05

**Authors:** Archana Radhakrishnan, Jennifer Henry, Kevin Zhu, Sarah T. Hawley, Brent K. Hollenbeck, Timothy Hofer, Daniela A. Wittmann, Anne E. Sales, Ted A. Skolarus

**Affiliations:** ^1^ Division of General Medicine University of Michigan Ann Arbor Michigan; ^2^ VA Health Service Research & Development Center for Clinical Management Research Ann Arbor Michigan; ^3^ Department of Health Management and Policy University of Michigan Ann Arbor Michigan; ^4^ Division of Oncology Department of Urology University of Michigan Ann Arbor Michigan; ^5^ Dow Division of Health Services Research Department of Urology University of Michigan Ann Arbor Michigan; ^6^ School of Social Work University of Michigan Ann Arbor Michigan; ^7^ Department of Learning Health Sciences University of Michigan Ann Arbor Michigan

**Keywords:** behavior change, cancer specialists, implementation science, primary care, quality, survivorship, theoretical domains framework (TDF)

## Abstract

**Background:**

With over 3 million US prostate cancer survivors, ensuring high‐quality, coordinated cancer survivorship care is important. However, implementation of recommended team‐based cancer care has lagged, and determinants of quality care across primary and specialty care remain unclear. Guided by the theoretical domains framework (TDF), we explored multidisciplinary determinants of quality survivorship care in an integrated delivery system.

**Methods:**

We conducted semistructured interviews with primary (4) and specialty (7) care providers across 6 Veterans Health Administration clinic sites. Using template analysis, we coded interview transcripts into the TDF, mapping statements to specific constructs within each domain. We assessed whether each construct was perceived a barrier or facilitator, examining results for both primary care providers (PCPs) and prostate cancer specialists.

**Results:**

Cancer specialists and PCPs identified 2 primary TDF domains impacting their prostate cancer survivorship care: *Knowledge* and *Environmental context and resources*. Both groups noted *knowledge* (about survivorship care) and *procedural knowledge* (about how to deliver survivorship care) as positive determinants or facilitators, whereas *resources*/*material resources* (to deliver survivorship care) was noted as a negative determinant or barrier to care. Additional domains more commonly referenced by cancer specialists included *Social/professional role and identity* and *Goals*, while PCPs reported the domain *Beliefs about capabilities* as relevant.

**Conclusions:**

We used the TDF to identify several behavioral domains acting as determinants of high‐quality, team‐based prostate cancer survivorship care. These results can inform prostate cancer survivorship care plan content, and may guide tailored, multidisciplinary implementation strategies to improve survivorship care across the primary and specialty care interface.

## 1 INTRODUCTION

Providing high‐quality cancer survivorship care is challenging. Not only are the number of cancer survivors rapidly growing, many older with several medical comorbidities, there is also an increasing oncologist shortage leading to an inability to meet the demands of the cancer survivor population.^1^, ^2^Nearly a quarter of cancer survivors have faced prostate cancer and many of these men have persistent urinary, sexual, bowel, and psychosocial symptoms, necessitating long‐term management similar to a chronic disease.^3^ While most men have follow‐up with both primary care providers (PCPs) and cancer specialists, which provider is responsible for delivering survivorship care is often unclear leading to gaps in quality prostate cancer survivorship care.^4^, ^5^, ^6^, ^7^, ^8^

Over a decade ago, the National Academies of Sciences released “From Cancer Patient to Cancer Survivor” calling for research on the determinants of high‐quality survivorship care across the primary and specialty care interface.^9^ Several strategies such as formal survivorship care plans and shared‐care models between primary and specialty care providers have been recommended; however, their success has been mixed.^10^, ^11^, ^12^, ^13^ One potential explanation rests upon a poor understanding of what primary and specialty care providers identify as drivers, or determinants, of high‐quality survivorship care. For example, PCPs might endorse a lack of knowledge in survivorship care, while oncologists report lack of time and resources to deliver this care.^14^, ^15^, ^16^ Indeed, optimizing survivorship care requires better understanding behavioral determinants acting as barriers and facilitators, and addressing those determinants through tailored, multidisciplinary interventions.

For these reasons, we explored prostate cancer survivorship care among PCPs and cancer specialists within an integrated healthcare delivery system. We used an innovative implementation research framework to characterize multidisciplinary determinants associated with quality care. Our approach to provider interviews informs survivorship care content and tailored interventions to support cancer specialists and PCPs to deliver quality prostate cancer survivorship care.

## 2 METHODS

### 2.1 Participant recruitment

We recruited providers from three different Veterans Health Administration (VHA) clinical sites within the Midwest region. We purposefully sampled participants from primary care, urology, medical oncology, and radiation oncology clinics to maximize variation in the sample and achieve a sample representative of the types of providers involved in prostate cancer survivorship care. We first contacted service chiefs to obtain permission to contact their providers. Once permission was obtained, an e‐mail was sent to providers that explained the study and gave them the option to opt‐out of participating. Providers were excluded if they had not provided care to at least 3 men with prostate cancer within the past year. This study was approved by the VA Ann Arbor Healthcare System Institutional Review Board.

### 2.2 Interview guide development

We developed our interview guide based on the theoretical domains framework (TDF) to understand determinants of provider behavior regarding prostate cancer survivorship care, and to inform future implementation strategies aimed at improving care across the primary and specialty care interface.^17^ The TDF uses constructs from over 30 psychological behavior change theories to assess barriers to practice change, and to inform the design of effective interventions based on those constructs acting as barriers and facilitators. There are 14 TDF domains (Knowledge, Skills, Social/professional role and identity, Beliefs about capabilities, Optimism, Beliefs about consequences, Reinforcement, Intentions, Goals, Memory, attention and decision processes, Environmental context and resources, Social influences, Emotion, and Behavioral regulation), each linked with evidence‐based behavior change techniques. Using this robust systematic approach to our interview guide development and to structure our qualitative findings is important because using TDF not only enables us to identify determinants of quality survivorship care across the primary and specialty care interface, but we can subsequently use these TDF determinants to direct selection of behavior change strategies and interventions most likely to address survivorship care gaps.^18^ For example, barriers endorsed by patients in the *Beliefs about capabilities* domain of TDF (eg, patient's belief regarding their PCP's capability to manage active surveillance) can be intervened upon by providing written or visual information to clarify provider roles and responsibilities. This may, in turn, improve the patient's professional confidence in their PCP to provide cancer care.

We designed our interview guide to assess several aspects of survivorship care including: (1) provider recognition of prostate cancer survivorship care (eg, monitoring prostate specific antigen [PSA] for recurrence, bone health for men on androgen deprivation therapy) and the benefits of survivorship interventions (eg, treatment of osteoporosis, incontinence, impotence); (2) the interface between PCPs and cancer specialists (eg, cancer specialty care availability) and survivorship care practice patterns; (3) behavioral control barriers to delivering survivorship care (eg, beliefs about capabilities); and (4) intention to perform prostate cancer survivorship care (see Appendix 2 for interview guide).

Eleven semi‐structured interviews were conducted by 2 members of the study team (JH and TS) and included 4 PCPs, 4 urologists, and 3 oncologists (2 radiation, 1 medical). No new major themes arose by the end of 11 interviews, implying that saturation had been reached. Based on the location and availability of the provider, we conducted 5 in‐person and 6 telephone interviews. All participants gave verbal consent prior to beginning the interview. Each interview began with a description of an index patient who was 1‐year postrobotic prostatectomy that the interviewee was told to keep in mind while responding to the interview questions. Interview questions probed the content areas highlighted above. Interviews were audio‐recorded, transcribed verbatim, and entered into NVivo software (NVivo, Version 11) for analysis.

### 2.3 Data analysis

We conducted data analysis in 2 steps. First, we mapped all content from each interview to a relevant TDF domain (KZ, JH, TS). Then, our research team (KZ, JH), including a prostate cancer specialist (TS) and primary care physician (AR) both with extensive survivorship care clinical and research expertise, mapped all TDF domain content to TDF constructs (see Appendix 3 for coding definitions). During this process, our research team collectively assessed whether the construct was perceived as a barrier (negative determinant) or facilitator (positive determinant) by the interviewee by rating responses within a range (−2 strong barrier, −1, 0, 1, 2 strong facilitator). Coding disagreements were resolved by group consensus, and we selected exemplar quotes where appropriate. We examined results both overall and separately by cancer specialists and PCPs using NVivo. This included an assessment of total references to TDF domains by PCPs and cancer specialists, and the valence of determinants across the range of barriers and facilitators for a given TDF domain.^19^

## 3 RESULTS

**Figure 1:**
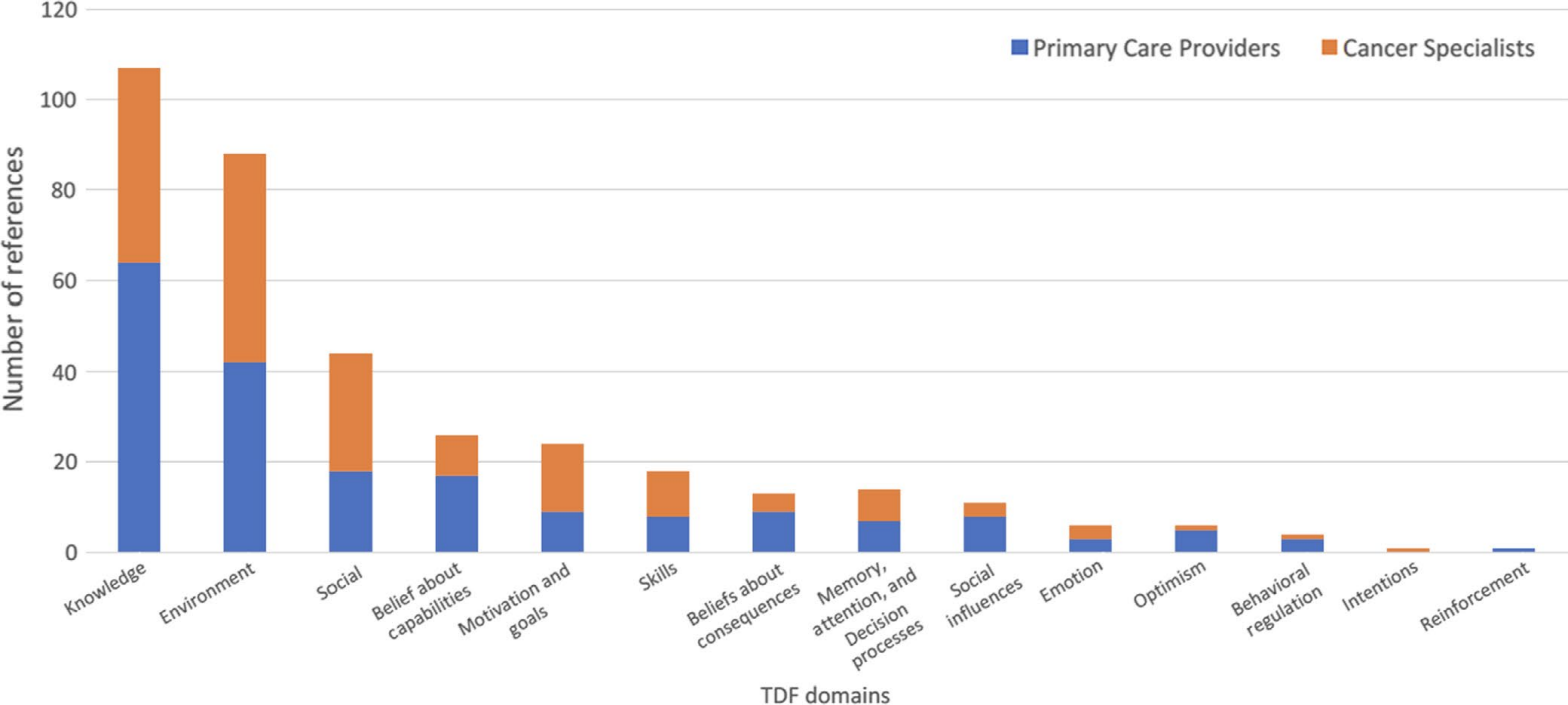
References to theoretical domains framework (TDF) domains by primary care providers and cancer specialists

We identified 2 primary domains impacting the multidisciplinary delivery of quality prostate cancer survivorship care: *Knowledge* and *Environmental context and resources*. These 2 domains accounted for the majority of all interview content, followed by *Social influences*,* Beliefs about capabilities*, and *Goals*, among others (Figure 1).

### 3.1 Knowledge

*Knowledge*, defined as the “awareness of the existence of something,” was the most frequently identified domain by all providers, referenced 64 times by PCPs and 43 times by cancer specialists (Appendix Table A1). Both cancer specialists and PCPs had general knowledge about prostate cancer survivorship care including assessing for treatment side effects and managing complications (eg, erectile dysfunction) and monitoring for recurrence (eg, serial PSA testing). However, knowledge barriers to survivorship care were also noted by both provider types. Cancer specialists reported not using formal survivorship care plans or not having them available within their clinics while PCPs reported lack of familiarity with or not receiving survivorship care plans. Both cancer specialists and PCPs also endorsed having *procedural knowledge* about how to deliver survivorship care, a construct within the domain of *Knowledge* (refer to Table 1 for example quotes). For example, cancer specialists reported referencing National Cancer Comprehensive Network guidelines for monitoring protocols and using standardized measures for symptom assessment (eg, International Prostate Symptom Score). On the other hand, PCPs endorsed using organizational resources such as electronic consults, a service available within the electronic medical record, to contact a cancer specialist about follow‐up on PSA tests on their mutual patient. One PCP noted, “Yeah, I mean e‐consults are I think a fabulous way of getting questions answered. You know it allows specialists to kind of lay out a detailed structure plan of things, you know plan a, and if you need to go to plan b, and c, so I think e‐consults for that purpose are great.”

**Table 1:** Summary of most commonly referenced TDF domains and constructs

Domain	Subdomain	PCPs		Cancer specialists	
		Summary	Example	Summary	Example
Knowledge	Knowledge	PCPs have knowledge about survivorship care, but rarely receive formal survivorship care plans or specific training or education.	“I've not seen specific survivorship treatment plans in terms of what that should look like or what that profile might look like. I think we're largely building our own you know based on the individual malignancy that we're taking care of.”	Specialists are knowledgeable about survivorship care but unfamiliar with formal survivorship care plans.	“What I have seen limiting survivorship care in general is just a lack of knowledge or lack of understanding of (a) what resources are available to somebody and (b) a lack of understanding of what survivorship care really means.”
	Knowledge of task environment	PCPs are aware of processes of care within their clinical contexts and know how to utilize resources available to deliver survivorship care.	“… people have ED, you have ED kind of service … so we refer people for that. Um … we're pretty familiar with Primary Care Mental Health you know and so people who have kind of symptoms … we'll send them to that …”	Specialists use their notes to track patient care and assist when transferring patients back to PCPs.	“… let's say I'm seeing patients for follow up and … I put … ‘Return to PCP’, and what is the plan of care, … ‘PSA once a year and alert Urology if PSA is rising or any other problem’, and again realistically … patients can schedule appointments themselves. So if let's say something happens … they can always do it, sort of initiate or re‐initiate follow up, things like that.”
	Procedural knowledge	PCPs are aware of how to treat prostate cancer patients, and communicate with specialists in a dynamic process.	“I use the e‐consults … I'll say you know” the PSA is up to this, is this okay or should I check it again quickly or do you guys want to see him?	Specialists are responsible for the patient's direct cancer care, and then transition the patient to primary care.	“I usually after 2 yr and they're having stable PSA, and they're comfortable with their outcomes, then we'll move to Primary Care and with recommendations of when they should come back to us.”
Environmental context and resources	Facilitators and barriers	Veterans receiving specialty care outside of the VA is a barrier to primary care treatment. Consulting Urology can be a barrier for PCPs.	“… but the biggest barrier is when we don't have that information … they were seeing a urologist on the outside, but now are transferring care here, so until we are able to get those results we are kind of lost about what to do.” “I mean one of the biggest barriers I have is about consulting Urology … some thought needs to go into what I'm presenting and giving a meaningful consultant response …”	Factors that affect communication between specialists and PCPs can be barriers or facilitators to treating patients.	“It's very helpful in terms of coordinating care if I know where their care is coming from and if I can communicate with the other physicians easily, and then things that hinder care are patients that don't stay within the system or kind of bounce around that can hinder an ability to get a sense of what the Primary Care doctor is doing.”
	Resources/material resources	Educational materials and/or tools would be helpful in clinical practice.	“… it would be nice to have kind of a go‐to brief education area … where you can say,” “this is what to expect when you're treating someone with prostate cancer who's had a prostatectomy or who's had radiation, you know these are the common things you're probably going to have to deal with …”	Time is a scarce resource and acts as a barrier to specialists.	“There's just no time. We barely have time to talk about their diabetes and their like new fracture, their growing prostate cancer let alone, I mean every other clinic I'm admitting someone to the hospital because of like some other life‐threatening thing so talking about like sexual dysfunction is just not kind of at the top of the radar.”
	Organizational culture/climate	PCPs have high caseloads and understand that specialty care should be reserved for patients who need that care	“The key is, is that primary care then needs to be supported with the correct amount of time, correct amount of patients, and correct amount of support staff.”	Positive working relationships with specialists facilitates best patient care practices.	“Having a good relationship with urology, medical oncology makes a big difference, even nuclear medicine for bone scans and things, it makes a big difference in really getting these patients where they need to be in a timely fashion and getting the answers that they need”
	Person × environment interaction	Co‐location of primary care and urology facilitates communication	“I think Urology is actually fairly good here about communicating with Primary Care maybe also because it's co‐located and I'm sure proximity helps right, so you can walk down the hall and talk with someone.”	An integrated healthcare system can facilitate care delivery (eg, communication between providers, access to resources).	“… I think most patients like to come for follow up to see their doctors about cancer care, to find out that everything is reassured, that things are going in the right track, so I think there are great benefits of providing that type of follow up.”
	Environmental stressors	Providers must consider the insurance coverage and cost to their patients.	“But for their office visit I'll ask like, ‘Do you get a bill from here, do you pay for coming here, do you pay for coming in here’? and sometimes it's also that they get only one bill depending upon several services they see on that day … so we say, ‘Okay, we'll try and coordinate it for you so that you get seen on the same day and you get charged only one co‐pay’.”	In order for survivorship care plans to be successful in VA, providers need more support.	“We need to have … people helping us in clinic … like a survivorship care person who's going to do all these survivorship care plans for all the patients and work with the physicians. So we can't have physicians now doing everything. It's just not sustainable, they need their, they're already burning out.”

ED, erectile dysfunction; TDF, theoretical domains framework; PCPs, primary care providers; PSA, prostate specific antigen.

### 3.2 Environmental context and resources

**Figure 2:**
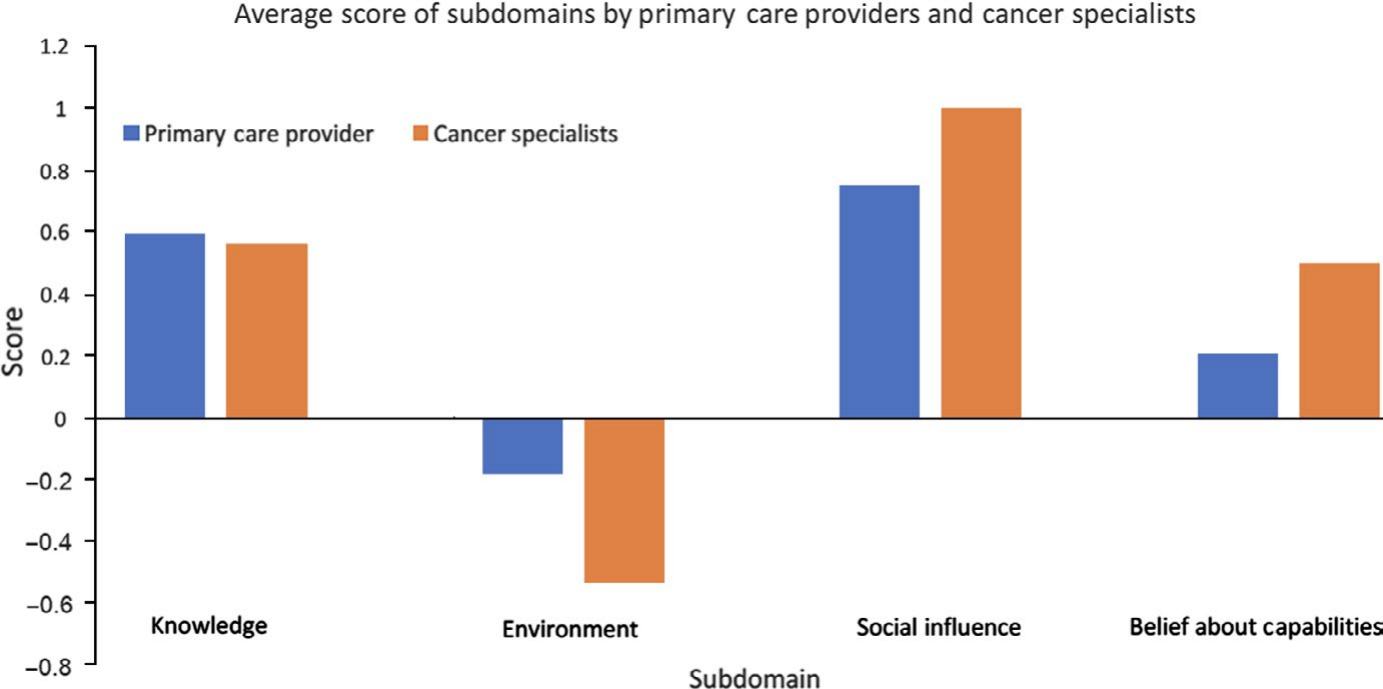
Perceptions of theoretical domains framework domains as positive determinants (facilitators) or negative determinants (barriers) to quality prostate cancer survivorship care according to provider type

Defined as “any circumstance of a person's situation or environment that discourages or encourages the development of skills and abilities, independence, social competence, and adaptive behavior,” environmental context and availability of resources were often noted by providers as barriers to delivering quality prostate cancer survivorship care (Figure 2). Specifically, the lack of *resources/material resources* was reported by several providers including: (1) lack of communication from cancer specialists regarding the standardized follow‐up care a patient needs (PCP noted, “it would be nice to have a summary of what all was the diagnosis … their Gleason score … what was the treatment … what all complications that the patient currently [is] having and … the current plan that's being done by Urology or radiation”); (2) lack of access to specialists (cancer specialist noted “… we have certain barriers currently … where if a patient does want to have treatment for bad incontinence …, we currently don't have a reconstructive surgeon …”); (3) lack of time during clinic visits to properly address all of the patient's concerns, especially in the context of other chronic conditions (cancer specialist stated, “There's just no time. We barely have time to talk about their new fracture from their growing prostate cancer let alone, I mean every other clinic I'm admitting someone to the hospital because of some other life‐threatening thing, so talking about sexual dysfunction is just not kind of at the top of that radar”); and 4) lack of support services for providers (eg, mental health services to address psychological concerns) and patients (eg, support groups).

In contrast, what providers reported as a facilitator to providing survivorship care involved the *organizational culture/climate*. Often, this was described as having:

… good relationships with urology, medical oncology … it makes a big difference in really getting these patients where they need to be in a timely fashion and getting the answers that they need because when they sit in your office and they're asking you questions that you can't necessarily deal with, it's very comforting that I can tell a patient, you know “I don't know that answer but I can go find out …”

In addition, the *person × environment interaction* was also noted as a facilitator to be able to deliver survivorship care. In other words, colocation of PCPs with cancer specialists was endorsed as facilitating communication between providers. As one PCP noted, “I think it's definitely helpful to be onsite, you can actually ask questions … It's not always that we know what we're doing, so it's kind of nice to curbside and ask …”

### 3.3 Comparison between PCPs and cancer specialists

Compared to cancer specialists, PCPs made more references to *Beliefs about capabilities* in their delivery of prostate cancer survivorship care (Appendix Table A1, 17 vs 9 references respectively for PCPs and cancer specialists). PCPs endorsed having *professional confidence* (an individual's belief in his or her repertoire of skills, and ability especially as it is applied to a task or set of tasks) in handling many aspects of follow‐up care for their patients and feeling comfortable doing so. One PCP noted, “… I think we try to manage them … most of the time probably. Primary Care does the majority of managing of the symptoms … and then for the ones that are really refractory we end up sending them back to urology, but I do feel kind of responsible for a pretty broad range.” Cancer specialists, on the other hand, reported *Social/professional role and identity* more frequently as relevant to their care (26 vs 18 references respectively for cancer specialists and PCPs). The majority of cancer specialists discussed feeling responsible for the patient's cancer control (ie, monitoring for recurrence) and assessing quality of life (eg, managing side effects from treatment). Cancer specialists varied in their views on sharing care with PCPs. One cancer specialist determined their continued involvement in their patient's care based on how involved the PCP was. But several others reported being involved in all aspects of their patient's survivorship care and even assuming primary care roles.

## 4 DISCUSSION

This study used the TDF to identify determinants of team‐based prostate cancer survivorship care within an integrated delivery system. Both PCPs and cancer specialists endorsed *Knowledge* (as a facilitator) and *Environmental context and resources* (as a barrier) as relevant to their survivorship care delivery. As the population of cancer survivors grows, understanding factors that influence provider abilities to deliver high‐quality survivorship care is critical. Increasingly, team‐based care models have been proposed to meet the diverse health needs of cancer survivors, however, how PCPs and cancer specialists deliver coordinated care have remained unclear. Our study helps clarify issues facing primary and specialty care and suggests directions forward to support them in their care for men surviving prostate cancer.

We found that *Knowledge* was the most frequent domain referenced by providers in this study, with both PCPs and cancer specialists endorsing having *knowledge* about prostate cancer survivorship care and perceiving it as a facilitator to delivering care. Prior studies have highlighted that PCPs often report lacking knowledge about survivorship care but also that cancer specialists lack confidence in PCPs’ abilities to do so.^20^, ^21^ There are several possible reasons for the differences noted in our study. First, providers endorsed having procedural knowledge, in other words, “knowing how to do something.” This is critical as PCPs have previously reported needing not only detailed plans for follow‐up care during survivorship but also having access to cancer specialists to ask questions.^22^, ^23^ Being within an integrated delivery system, such as the VHA, may facilitate this and interventions that leverage similar resources, such as universal access to electronic medical records and electronic consults to improve communication between providers, will be important. Second, VHA largely consists of male patients, making prostate cancer and its sequelae more common, thereby adding to PCP expertise. Third, the majority of prostate cancer in this population is localized limiting the scope of survivorship care. For example, compared to pediatric malignancies where screening for secondary malignancies and repetitive imaging are common, the long‐term and late effects of definitively treated localized prostate cancer among older men may be more straightforward.^24^ Leveraging knowledge as a facilitator to providing survivorship care, especially by PCPs, will be instrumental moving forward in designing strategies to increase PCP involvement and transition survivorship care from the cancer specialist to the PCP.

Quality survivorship care delivery requires both time and resources, and this was a barrier frequently reported as negatively impacting clinical practice. As increasing calls to improve cancer survivorship care delivery have been made over the past decade, policy changes at various levels (organizational, national) to facilitate implementation of efficient and effective survivorship care programs are needed.^25^ This becomes more relevant as provision of survivorship care plans is now a quality metric used in cancer center accreditation, placing the burden primarily on cancer specialists and their teams.^26^ This was supported by our findings attributing stronger negative determinants to the *Environment* domain among cancer specialists. Additionally, in an example of an intervention implemented to improve survivorship care, resources specifically included dedicated staff members to complete survivorship care plans, an oncology nurse practitioner to review treatment summaries and recommendations, and a social worker to address late‐ and long‐term psychosocial effects.^27^ This model of care led to comprehensively addressing physical and psychosocial effects from treatment and high patient satisfaction. Coupled with our work, these findings indicate addressing resource needs for survivorship care is critical to optimize survivorship care models in and outside of this system.

One key challenge to team‐based survivorship care models is a lack of clarity among providers regarding responsibility for survivor follow‐up care. Results from our study highlight the discrepancy between cancer specialists and PCPs on their respective roles. While some cancer specialists perceived their roles as extending to addressing primary care needs, PCPs reported feeling comfortable and having confidence in managing their patient's prostate cancer follow‐up care. This suggests that improving care coordination between cancer specialists and PCPs requires clear delineation of responsibilities for what each provider will handle, and this ideally needs to be communicated to patients. For example, strategies, such as web‐based patient tools that describe team‐based models of survivorship care and specific roles for cancer specialists and PCPs, can be helpful in accomplishing this.

This study has some limitations. First, because we were able to achieve thematic saturation with 11 providers, it is likely that we identified the two key domains necessary for quality survivorship care. In fact, our findings are consistent with others regarding resources as a determinant of survivorship care plan use.^19^ While we were able to achieve granularity in understanding factors that impact primary and specialty care providers’ daily clinical practices, and identify domains and constructs as potential targets for future interventions to improve survivorship care, further work is needed to understand how best to effectively address those determinants in clinical practice. Second, our providers were from the VHA, which is an integrated delivery system where providers have universal access to electronic medical records. While this may not be fully generalizable to other care settings, it represents an important case scenario on how to coordinate care at the primary and specialty care interface especially given increasing electronic record exhange across health systems. Third, while we used TDF to guide our interviews, it is possible that some domains were not represented. For example, the importance of “communication” between cancer specialists and PCPs was mentioned in several cases with one cancer specialist noting, “It's very helpful in terms of coordinating care if I can communicate with the other physicians easily …” while a PCP reported as a problem not receiving medical records regarding patient treatment from providers outside of their medical system. While our coding using the TDF classified these as barriers (within Environmental resources/context domain) and knowledge (within Knowledge domain), a more accurate classification might be “communication.” Nonetheless, evidence‐based behavior change strategies within these domains targeting increased communication among providers would appear valid (ie, supporting communication of survivorship care plans or outside medical records). Overall, the rigorous development and validation of this behavioral framework along with its ties to evidence‐based behavior change techniques make it an excellent tool for dissecting survivorship care practices and directing future efforts to improve care.^18^, ^28^, ^29^ In addition, while our quantification of references to TDF domains and constructs has limitations, the relative relationships among the domains in terms of relevance to survivorship care intervention development is an important take‐away message. For example, interventions might consider targeting the leading domains rather than those infrequently referenced (eg, emotion, intention) as the focus of changing behavior with respect to primary and specialty survivorship care.

PCPs and cancer specialists identified several constructs within the TDF domains as relevant to their prostate cancer survivorship care delivery. While knowledge about survivorship care was perceived as a facilitator, limited resources to be able to deliver survivorship care was reported as a barrier. Our results provide critical insight into factors that providers perceive as being important in their clinical practices. These behavioral theory‐based results may inform future efforts in the design and implementation of prostate cancer survivorship care plan content, and guide tailored, multidisciplinary implementation strategies to improve prostate cancer survivorship care across the specialty and primary care interface.

## CONFLICTS OF INTEREST

Dr. Wittmann is funded by the Movember Foundation and Dr. Skolarus’ R37 CA222885 from the National Cancer Institute.

## APPENDIX 1

### 1.1

**Table A1:** Number of references to theoretical domain framework (TDF) constructs for prostate cancer survivorship care according to provider type

TDF domain with constructs	All interviewees	Primary care providers	Cancer specialists
Behavioral regulation	4	3	1
Action planning	2	1	1
Breaking habit	0	0	0
Self‐monitoring	2	2	0
Beliefs about capabilities	26	17	9
Beliefs	0	0	0
Empowerment	0	0	0
Perceived behavioral control	6	3	3
Perceived competence	4	1	3
Professional confidence	11	9	2
Self‐confidence	4	3	1
Self‐efficacy	2	1	1
Self‐esteem	0	0	0
Beliefs about consequences	13	9	4
Anticipated regret	1	1	0
Beliefs	5	2	3
Characteristics of outcome expectancies	0	0	0
Consequents	3	3	0
Outcome expectancies	5	3	2
Emotion	6	3	3
Affect	0	0	0
Anxiety	0	0	0
Burn‐out	0	0	0
Depression	1	0	1
Fear	1	0	1
Positive/negative affect	1	1	0
Stress	2	1	1
Environmental context and resources	88	42	46
Barriers and facilitators	19	7	12
Environmental stressors	4	3	1
Organizational culture/climate	15	7	8
Person × environment interaction	9	5	4
Resources/material resources	44	21	23
Salient events/critical incidents	0	0	0
Intentions	1	0	1
Stability of intentions	1	0	1
Stages of change model	0	0	0
Transtheoretical model and stages of change	0	0	0
Knowledge	107	64	43
Knowledge of task environment	13	8	5
Knowledge	57	34	23
Procedural knowledge	36	21	15
Memory, attention, and decision processes	14	7	7
Attention	7	5	2
Attention control	2	0	2
Cognitive overload or tiredness	0	0	0
Decision‐making	5	2	3
Memory	0	0	0
Goals	24	9	15
Action planning	0	0	0
Goal—target setting	0	0	0
Goal priority	4	2	2
Goals—autonomous or controlled	0	0	0
Goals—distal or proximal	2	2	0
Implementation intention	1	1	0
Optimism	6	5	1
Identity	0	0	0
Optimism	2	2	0
Pessimism	3	2	1
Unrealistic optimism	1	1	0
Reinforcement	1	1	0
Consequents	0	0	0
Contingencies	0	0	0
Incentives	0	0	0
Punishment	0	0	0
Reinforcement	1	1	0
Rewards	0	0	0
Sanctions	0	0	0
Skills	18	8	10
Ability	1	1	0
Competence	4	2	2
Interpersonal skills	4	2	2
Practice	0	0	0
Skill assessment	4	2	2
Skills development	0	0	0
Skills	3	1	2
Social influences	11	8	3
Alienation	1	1	0
Group conformity	1	1	0
Group identity	0	0	0
Group norms	0	0	0
Intergroup conflict	0	0	0
Modeling	0	0	0
Power	0	0	0
Social comparisons	1	1	0
Social norms	3	2	1
Social pressure	4	2	2
Social support	1	1	0
Social/professional role and identity	44	18	26
Group identity	3	1	2
Identity	0	0	0
Leadership	0	0	0
Organizational commitment	0	0	0
Professional boundaries	9	4	5
Professional confidence	3	1	2
Professional identity	3	1	2
Professional role	30	12	18
Social identity	0	0	0

## APPENDIX 2 INTERVIEW GUIDE FOR PROVIDER SEMISTRUCTURED INTERVIEW

### 2.1

**Time**: 45 min

### Introduction

Is this still a good time for you? Are you in a place where you can be free from distractions and feel free to give candid responses? Would it be OK with you if I record this call? [If they ask why, say for research and training purposes.]

Thank you for agreeing to participate in this interview. The aim of the study is to help us understand improved prostate cancer survivorship care by learning more about it. As a provider, you serve as a primary source of information and will be able to provide us with valuable information. During this interview, I will ask about your behavior and perspective on survivorship care of prostate cancer patients.

Your responses will help to inform conclusions regarding the appropriate role of various specialists in survivorship care. All of your responses will remain confidential and will only be reported in aggregate. You may choose to stop the interview at any time, and there is no penalty to you or your organization for not completing the interview.

Do you have any questions before we begin?

**Index patient**: Sixty‐eight‐year‐old male status postrobotic prostatectomy 1 year ago with urine leakage (two pads per day) and erectile dysfunction.

### Interview

**Let's start with some general questions about survivorship care and then move into your specific involvement.**

In a few sentences can you describe the role of a (PCP, urologist, radiation and medical oncologist) in the survivorship of patients with prostate cancer?

If one of your patients has prostate cancer, what aspects of his survivorship care do you feel personally responsible for?

What do you consider to be the most fundamental aspects of quality survivorship care for a patient with prostate cancer?

What is the purpose of prostate cancer survivorship care?

Is survivorship care part of your job as a (PCP, urologist, radiation, or medical oncologist)?

Can you tell me how personally involved you are in the survivorship care of your prostate cancer patients?

How much personal experience do you have in survivorship care?

Do you believe it should be part of your job?

Is survivorship care consistent amongst your practice? Hospital?

Have you received training that is specific to providing survivorship care?

Walk me through the steps you take in planning/carrying out survivorship care.

- Prompts: Does it depend? If so, what does it depend on? Does the stage change your plan? The patient's comfort level? Life expectancy? Severity of pain? Cost of care? Patient satisfaction? Peer behavior? Possible consequences? Which of these do you consider most important?

What do you consider to be your most frequently used intervention method for prostate cancer survivorship care?

- Prompts: Androgen deprivation therapy (ADT) injections, prostate specific antigen (PSA) monitoring, monitoring bone health, treatment of osteoporosis, incontinence, impotence, etc.

From your perspective, what are the main barriers and facilitators to you providing quality survivorship care?

- Prompts: What specifically helps you or hinders you? What encourages you? For example, reminders, incentives. Which of these helps you most?

Do you feel you have adequate access to all cancer specialty care resources?

- Prompts: Do other specialties have resources you do not have?

In what way does your specific facility enable or inhibit your survivorship care?

What kind of patients will you specifically take on to provide survivorship care?

- Prompts: What kind of patients will you not take? Who assumes care at that point and why? Does it depend on the situation?

Would you feel obliged as a (PCP, urologist, medical oncologist) to assume survivorship care for the index patient?

Would you feel completely comfortable assuming care for the index patient?

How optimistic would you feel when treating the index patient?

- Prompts: Do you usually expect the best? Are you always optimistic about the future?

Does the amount of time you have influence your decision to provide survivorship care?

What things do you usually do in preparation for longitudinal survivorship care?

- Prompts: Do you discuss the treatment plan with the patient? Do you review the literature? Consult colleagues? Schedule appointments?

Do patients see a (PCP, urologist, medical oncologist) each time they have an appointment?

In what ways do your feelings influence your care?

- Prompts: For example, if you feel anxious about the patient's situation are you likely to act differently?

How do you follow such patients’ PSA values?

- Prompts: Every 3, 9, 16 months?

Do many of your patients receive ADT injections?

- Prompts: What sort of patients receive ADT injections?

How much experience with ADT?

What is the purpose of ADT?

How do patients feel on ADT?

What are the side effects you are concerned about with ADT?

During the past 2 months, do you feel the outcomes of your survivorship patients have affected your day‐to‐day life more than other patients?

How do patients typically feel about their care from you?

What do you consider the benefits to the patient to be of following with you?

- Prompts: Would the benefits be similar with a (other specialist).

What are the expectations, requirements, and costs for your survivorship patients?

- Prompts: For example, time taken away from other tasks, need for occasional treatment/procedure, stress of PSA results, out of pocket costs, etc.

Have these factors ever affected your decision to follow a patient?

Do you that feel the benefits of your care outweigh the costs?

- Prompts: How so?

How important is it to you that your patient population consists of prostate cancer survivorship patients?

- Prompts: How much do you want to do it? Do you feel you are best suited? Are you compelled to do it? Are there other tasks that you perform in your job that are more important? Why?

Approximately how many patients will you offer to provide survivorship care to in the next 2 months?

- Prompts: How strong is this intention?

Have you ever forgotten about certain survivorship care options when treating patients?

- Prompts: Why do you think that is? Are there certain systems you could implement to prevent this in the future? Do you think a (PCP, urologist, medical oncologist) would have forgotten that aspect?

**Let's talk about opinions and what people in your clinical team think about survivorship care.**

In your opinion, how much does providing survivorship care to prostate cancer patients align with what somebody in your position should be doing?

What influential individuals or groups are in favor of (PCPs, urologists, medical oncologists) providing majority survivorship care?

- Prompts: Please tell me about them and their perspectives. Prompts: For example, clinical leaders, management, patients, top researchers etc.

Do you think about the opinions of these influential people when considering whether to take on a patient?

Do you feel that most people whose opinion you value would approve of you providing majority survivorship care to the index patient?

- Prompts: If you got the sense that others did not approve, would that influence whether or how you provide care?

If you sensed that your decision damaged your relationships in any way (with patients, other providers) would you be likely to change your actions?

Do you feel motivated in general to provide survivorship care?

- Prompts: Does this motivation level affect the likelihood of you providing care or not?

### Conclusion

That's all the questions I have for you, has anything occurred to you about this topic that I haven't asked about?

## APPENDIX 3 CODING OF THEORETICAL DOMAINS FRAMEWORK CONSTRUCTS

**Table:**

Domains	Constructs
(1) Knowledge (an awareness of the existence of something)	Knowledge: an awareness of the existence of something
	Procedural knowledge: knowing how to do something
	Knowledge of task environment: knowledge of social and material context in which task undertaken
(2) Skills (an ability or proficiency acquired through practice)	Skills: an ability or proficiency acquired through training and/or practice
	Skills development: repetition of an act, behavior, or series of activities, often to improve performance or acquire a skill
	Competence: one's repertoire of skills and ability especially as it is applied to a task or set of tasks
	Ability: competence or capacity to perform a physical or mental act. Ability may be either unlearned or acquired by education and practice
	Interpersonal skills: an aptitude enabling a person to carry on effective relationships with others, such as an ability to cooperate, to assume appropriate social responsibilities or to exhibit adequate flexibility
	Practice: repetition of an act, behavior, or series of activities, often to improve performance or acquire a skill
	Skill assessment: a judgment of the quality, worth, importance, level, or value of an ability or proficiency acquired through training and practice
(3) Social/professional role and identity (a coherent set of behaviors and displayed personal qualities of an individual in a social or work setting)	Professional identity: the characteristics by which an individual is recognized relating to, connected with, or befitting a particular profession
	Professional role: the behavior considered appropriate for a particular kind of work or social position
	Social identity: the set of behavioral or personal characteristics by which an individual is recognizable (and portrays) as a member of a social group
	Identity: an individual's sense of self defined by (a) a set of physical and psychological characteristics that is not wholly shared with any other person and (b) a range of social and interpersonal affiliations (eg, ethnicity) and social roles
	Professional boundaries
	Professional confidence: an individual's belief in his or her repertoire of skills, and ability especially as it is applied to a task or set of tasks
	Group identity: the image of a group (eg, reputation, appraisal, expectations about) held by its members or by those external to the group; an individual's sense of self as defined by group membership
	Leadership: the processes involved in leading others, including organizing, directing, coordinating, and motivating their efforts toward achievement of certain group of organization goals
	Organizational commitment: a distinctive pattern of thought and behavior shared by members of the same organization and reflected in their language, values, attitudes, beliefs, and customs
(4) Beliefs about capabilities (acceptance of the truth, reality, or validity about an ability, talent, or facility that a person can put to constructive use)	Self‐confidence: self‐assurance or trust in one's own abilities, capabilities and judgment
	Perceived competence: an individual's belief in his or her ability to learn and execute skills
	Self‐efficacy: an individual's capacity to act effectively to bring about desired results, as perceived by the individual
	Perceived behavioral control: authority, power, or influence over events, behaviors, situations, or people
	Beliefs: the thing believed; the proposition or set of propositions held true
	Self‐esteem: degree to which the qualities and characteristics contained in one's self‐concept are perceived to be positive
	Empowerment: the promotion of the skills, knowledge, and confidence necessary to take great control of one's life as in certain educational or social schemes; the delegation of increased decision‐making powers to individuals or groups in a society or organization
	Professional confidence: an individual's belief in his or her repertoire of skills, and ability especially as it is applied to a task or set of tasks
(5) Optimism (the confidence that things will happen for the best or that desired goals will be attained)	Optimism: attitude that outcomes will be positive and that people's wishes or aims will ultimately be fulfilled
	Pessimism: attitude that things will go wrong and that people's wishes or aims are unlikely to be fulfilled
	Unrealistic optimism: return or recompense made to, or received by a person contingent on some performance
	Identity: an individual's sense of self defined by (a) a set of physical and psychological characteristics that is not wholly shared with any other person and (b) a range of social and interpersonal affiliations (eg, ethnicity) and social roles
(6) Beliefs about consequences (acceptance of the truth, reality, or validity about outcomes of a behavior in a given situation)	Beliefs
	Outcomes expectancies: cognitive, emotional, behavioral, and affective outcomes that are assumed to be associated with future or intended behaviors. These assumed outcomes can either promote or inhibit future behaviors
	Characteristics of outcome expectancies: characteristics of the cognitive, emotional, and behavioral outcomes that individuals believe are associated with future or intended behaviors and that are believed to either promote or inhibit these behaviors.
	Anticipated regret: a sense of the potential negative consequences of a decision that influences the choice made
	Consequents
(7) Reinforcement (increasing the probability of a response by arranging a dependent relationship, or contingency, between the response and a given stimulus)	Rewards (proximal/distal, valued/not valued, probable/improbable)
	Incentives: an external stimulus, such as condition or object, that enhances or serves as a motive for behavior
	Punishment: the process in which the relationship between a response and some stimulus or circumstance results in the response becoming less probable; a painful, unwanted, or undesired event or circumstance imposed as a penalty on a wrongdoer
	Consequents: an outcome of a behavior in a given situation
	Reinforcement: the process in which the frequency of a response is increased by a dependent relationship or contingency with a stimulus
	Contingencies
	Sanctions: a punishment or other coercive measure, usually administered by a recognized authority, that is used to penalize and deter inappropriate or unauthorized actions
(8) Intentions (a conscious decision to perform a behavior or a resolve to act in a certain way)	Stability of intentions: ability of one's resolve to remain in spite of disturbing influences
	Stages of change model: a model that proposes that behavior change is accomplished through five specific stages: precontemplation, contemplation, preparation, action, maintenance
	Transtheoretical model and stages of change: a model that proposes that behavior change is accomplished through five specific stages: precontemplation, contemplation, preparation, action, maintenance
(9) Goals (mental representations of outcomes or end states that an individual wants to achieve)	Goals (distal/proximal): Distal: ultimate level of performances to be achieved. Proximal: preliminary levels of performances to be achieved while working toward distal
	Goal priority: order of importance or urgency of end states toward which one is striving
	Goal/target setting: process that establishes specific time‐based behavior targets that are measurable, achievable, and realistic.
	Goals (autonomous/controlled): assuredness of one's resolve to act in a certain way
	Action planning: the action or process of forming a plan regarding a thing to be done or a deed
	Implementation intention: the plan that one creates in advance of when, where, and how one will enact a behavior
(10) Memory, attention, and decision processes (the ability to retain information, focus selectively on aspects of the environment and choose between two or more alternatives)	Memory: the ability to retain information or a representation of past experience, based on the mental processes; specific information or a specific past experience that is recalled
	Attention: Focus on certain aspects of the environment rather than on others
	Attention control: action selection is held to be controlled by choices between routine functions that are performed automatically and nonroutine situations involving decision‐making
	Decision‐making: cognitive processes of choosing between two or more alternatives, ranging from the relatively clear cut to the complex
	Cognitive overload/tiredness: the situation in which the demands placed on a person by mental work are greater than a person's mental abilities
(11) Environmental context and resources (any circumstance of a person's situation or environment that discourages or encourages the development of skills and abilities, independence, social competence, and adaptive behavior)	Environmental stressors: External factors that requires one to change in some way (causing stress); stressors that are found in our surroundings
	Resources/material resources: Assets that can be utilized to function effectively
	Organizational culture/climate: A system of shared assumptions, values, and beliefs, which governs how people behave. Dictate how they perform their jobs
	Salient events/critical incidents: Most important, noticeable
	Person × environment interaction: The properties of the environment (benefits, reinforcers, satisfiers, payoffs) that correspond to the desires of the person (abilities, demands); match between individuals and environments (congruence, fit)
	Barriers and facilitators: in psychological contexts barriers/facilitators are mental, emotional, or behavioral limitations/strengths in individuals or groups
(12) Social influences (those interpersonal processes that can cause individuals to change their thoughts, feelings, or behaviors)	Social pressure: The exertion of influence on a person or group by another person or group. [like Group Pressure, social pressure include rational argument and persuasion, calls for conformity. Demands, threats, personal attacks, rewards, social approval]
	Social norms: any of the socially determined consensual standards that indicate what behaviors are considered typical in a given context and what behaviors are considered proper in the context
	Group conformity
	Social comparisons: people evaluate their abilities and attitudes in relation to those of others
	Group norms: See Social Norms
	Social support: the provision of assistance or comfort to others
	Power: the capacity to influence others
	Intergroup conflict: disagreement or confrontation between two or more groups and their members
	Alienation: estrangement from one's social group; a deep‐seated sense of dissatisfaction with one's personal experiences that can be a source of lack of trust in one's social or physical environment or in oneself; the experience of separation between thoughts and feelings
	Group identity: the image of a group held by its members or by those external to the group; an individual's sense of self as defined by group membership
	Modeling: learning occurring through observation and imitation
(13) Emotion (a complex reaction pattern, involving experiential, behavioral, and physiological elements, by which the individual attempts to deal with a personally significant matter or event)	Fear: an intense emotion aroused by the detection of imminent threat, involving an immediate alarm reaction that mobilizes the organism by triggering a set of physiological changes
	Anxiety: a mood state characterized by apprehension and somatic symptoms of tension in which an individual anticipates impending danger, catastrophe, or misfortune
	Affect: an experience or feeling of emotion, ranging from suffering to elation, from the simplest to the most complex sensations of feelings, and from the most normal to the most pathological emotional reactions
	Stress: a state of physiological or psychological response to internal or external stressors
	Depression: a mental state that presents with depressed mood, loss of interest or pleasure, feelings of guilt or low self‐worth, disturbed sleep or appetite, low energy, and poor concentration
	Positive/negative affect: the internal feeling/state that occurs when a goal has/has not been attained, a source of threat has/has not been avoided, or the individual is/is not satisfied with the present state of affairs
	Burn‐out: physical, emotional or mental exhaustion, especially in one's job or career, accompanied by decreased motivation, lowered performance, and negative attitudes towards oneself and others
(14) Behavioral regulation (anything aimed at managing or changing objectively observed or measured actions)	Self‐monitoring: a method used in behavioral management in which individuals keep a record of their behavior, especially in connection with efforts to change or regulate the self
	Breaking habit: to discontinue a behavior or sequence of behaviors that is automatically activated by relevant situational cues
	Action planning: the action or process of forming a plan regarding a thing to be done or a deed
